# Mortality trends in heart failure and colon cancer: Insights into gender, ethnic, and regional disparities in the United States (1999–2020)

**DOI:** 10.1016/j.ahjo.2025.100699

**Published:** 2025-12-16

**Authors:** Hafsah Alim Ur Rahman, Nimrah Iqbal, Muhammad Ahmed Ali Fahim, Fayza Salman, Syed Hassan Ahmed, Omama Asim, Taha Mansoor, Muhammad Zain Farooq, Muhammad Sohaib Asghar

**Affiliations:** a Dow University of Health Sciences, Karachi, Pakistan; b Western Michigan University Homer Stryker M. D. School of Medicine, Kalamazoo, MI, USA; c Moffitt Cancer Centre, FL, USA; d AdventHealth Sebring, FL, USA

**Keywords:** Colon cancer, Heart failure, Demographics, Age-adjusted mortality rates

## Abstract

**Background:**

Heart failure (HF) and colorectal cancer (CRC) are major public health concerns among the aging population in the United States. This study aimed to investigate temporal, regional, urbanization and racial trends in mortality among adults with HF and CRC aged ≥65 years.

**Methods:**

Mortality data were sourced from the Centers for Disease Control and Prevention Wide-Ranging Online Data for Epidemiologic Research (CDC WONDER) database, utilizing ICD-10 codes to identify deaths related to colon cancer and heart failure from 1999 to 2020. Age-adjusted mortality rates (AAMRs) per 100,000 individuals were calculated, along with Annual Percentage Changes (APCs) and their respective 95 % confidence intervals (CIs).

**Results:**

The AAMRs remained relatively stable between 1999 (8.5) and 2004 (7.3) (APC: −2.61; 95 % CI: −3.86, 0.09). From 2004 to 2009, a significant decline to 5.0 was observed (APC: −7.08; 95 % CI: −9.28, −3.58). Subsequently, the rates stabilized by 2015 (3.8) (APC: −4.84; 95 % CI: −6.58 to 2.04) but demonstrated a modest increase to 4.4 by 2020 (APC: 2.55; 95 % CI: 0.08 to 8.19). Mortality rates were consistently higher among males (6.7 vs. 4.5 for females) and varied across racial/ethnic groups, with Non-Hispanic (NH) Whites (5.7) and NH Black/African Americans (5.4) exhibiting the highest rates, while Hispanics (2.8) and NH Asians/Pacific Islanders (2.3) had the lowest. Regional disparities showed that the Midwest had the highest AAMRs (6.5) followed by the Northeast (5.4), West (5.2), and South (4.8). Additionally, non-metropolitan areas exhibited significantly higher rates than metropolitan areas (7.1 vs. 5.0, respectively). The states in the 90th percentile for AAMRs were West Virginia, Mississippi, South Dakota, Nebraska, and North Dakota.

**Conclusion:**

Although there was an overall decline in mortality rates during the study period, disparities remained evident, with higher mortality observed among males, non-Hispanic Whites, residents of the Midwest, and individuals in non-metropolitan areas. This highlights the need for targeted public health intervention.

## Graphical abstract

**Figure:**
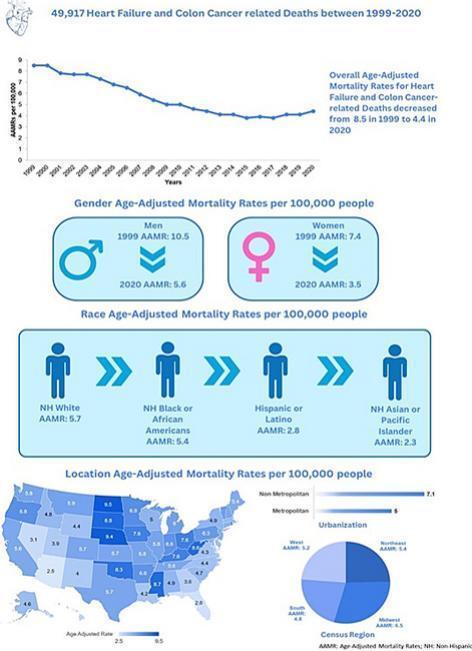

## 1 Introduction

Heart failure (HF) and colorectal cancer (CRC) represent two of the most pressing public health challenges affecting the older adults in the United States [1,2]. HF is a chronic, progressive condition marked by the inability of the heart to pump blood efficiently, leading to significant clinical outcomes such as diminished quality of life, recurrent hospitalizations, and elevated mortality rates [3]. Notably, the incidence of HF-related deaths has been increasing since 2012, with the highest prevalence observed among individuals aged ≥65 years [4,5]. CRC is among the most frequently diagnosed cancers in older adults and is a leading cause of cancer-related mortality. Despite advancements in screening and treatment, the incidence of CRC remains high, especially among seniors, with advanced-stage diagnoses often resulting in a poor prognosis [6].

This study examined HF and CRC concurrently, owing to several overlapping risk factors and pathophysiological mechanisms of both conditions, associated with chronic systemic inflammation and metabolic disorders, including obesity, diabetes, and hypertension [7]. Furthermore, emerging evidence highlights the cardiotoxic effects of certain cancer treatments, such as chemotherapy and radiation, which may elevate the risk of HF in CRC patients [8]. These shared risk profiles suggest a potential interplay between these two diseases, particularly in an aging population. To provide a comprehensive epidemiological analysis and enhance the understanding of the cumulative health burden of HF and CRC, this study analyzed age-adjusted mortality rates (AAMRs) and temporal trends among U.S. adults aged 65 years and older from 1999 to 2020.

This analysis utilized publicly available mortality data from the Centers for Disease Control and Prevention Wide-Ranging Online Data for Epidemiologic Research (CDC WONDER) Database, with a focus on sex-specific and geographic differences over time. By identifying significant trends and disparities in mortality rates, this study aims to propose effective public health strategies, improve population-level healthcare policies, and ultimately contribute to the reduction of preventable deaths and enhancement of health outcomes in elderly populations.

## 2 Methods

### 2.1 Population and study setting

This study employed the Centers for Disease Control and Prevention Wide-Ranging Online Data for Epidemiologic Research (CDC WONDER) database to examine mortality associated with HF and CRC among adults aged 65 years and older from 1999 to 2020. Data were extracted using the International Classification of Diseases, Tenth Revision (ICD-10) codes I50 for HF and C18–20 for CRC. Analysis was conducted on Data from death certificates across all 50 states and the District of Columbia, focusing on individuals aged 65 years or above at the time of death. Institutional review board approval was not required as the study utilized de-identified public data. This study adhered to STROBE standards for reporting observational research. The CDC WONDER database is instrumental in analyzing mortality trends related to HF and CRC. Ethnicities were specified as Non-Hispanic (NH) White, NH Black or African American, Hispanic or Latino, and NH Asian or Pacific Islander. This racial classification, in compliance with US Office of Management and Budget (OMB) guidelines, has been used in prior studies of the WONDER database [9]. Older adults were defined as individuals aged 65 years and above [2,5].

### 2.2 Data abstraction

The dataset encompasses population figures, year of occurrence, place of death, demographic details, geographical breakdowns, state-specific information, and distinctions between urban and rural settings. Deaths occur in various settings, including hospitals, homes, hospices, nursing homes, and long-term care facilities. The demographic data included sex, age, race, and ethnicity. Data sourced from death certificates have been utilized in previous studies that employed the WONDER database [10]. Population classification followed the National Center for Health Statistics Urban-Rural Classification Scheme, with urban areas comprising large metropolitan areas (≥1,000,000), medium/small metropolitan areas (50,000 to 999,999), and rural areas with fewer than 50,000 people per 2013 U.S. Census [11]. Geographically, the Northeast, Midwest, South, and West were the four main regions based on the U.S. Census Bureau criteria [12].

### 2.3 Statistical analysis

Age-adjusted mortality rates (AAMRs) per 100,000 individuals were calculated using the US 2000 standard population as a reference, ensuring compatibility across different years and demographic subgroups. Mortality rates were stratified by sex, race/ethnicity, place of death, geographic region, and urban-rural status, and analyzed annually with a corresponding 95 % confidence interval (CI). The Joinpoint Regression Program (version 5.02, National Cancer Institute) was used to identify significant trends over time, along with a log-linear model to detect statistically significant shifts in mortality trends. The annual percent change (APC) and its 95 % CI in AAMRs were calculated to evaluate the changes in mortality rates for heart failure and colon cancer from 1999 to 2000. The log-linear model classified APCs as either increasing or decreasing based on a significance level of *p* < 0.05. Additionally, the stability of mortality trends during different time intervals was assessed to explore periods of significant decline or increase.

## 3 Results

From 1999 to 2020, heart failure and colon cancer were responsible for 49,917 deaths in patients aged ≥65 years (Supplementary Table 1). The overall AAMR for all age groups was 5.4 (95 % CI: 5.4–5.5). When stratified by place of death, medical facilities accounted for 35.99 % of deaths, nursing homes for 27.22 % of decedents, and hospices for 3.81 %. Moreover, 4.12 % of deaths occurred in places that could not be classified into the aforementioned categories, with the place of death for the remaining 69 patients marked unknown (Supplementary Table 2). The demographic data are provided in Table 1.

**Table 1:** Demographic characteristics of deaths due to heart failure and colon cancer in older adults in the United States, 1999 to 2020.

Variable	Heart failure and colon cancer deaths n (%)	AAMRs (95 % CI) per 100,000
Overall population	49,917 (100)	5.4 (5.4–5.5)
Sex		
Male	23,615 (47.31)	6.7 (6.6–6.8)
Female	26,302 (52.69)	4.5 (4.5–4.6)
Census region		
Northeast	10,220 (20.47)	5.4 (5.3–5.5)
Midwest	13,914 (27.87)	6.5 (6.4–6.6)
South	15,802 (31.66)	4.8 (4.7–4.9)
West	9981 (20.00)	5.2 (5.1–5.3)
Race/ethnicity		
NH American Indian or Alaska Native	167 (0.33)	–
NH Asian or Pacific Islander	694 (1.39)	2.3 (2.1–2.4)
NH Black or African American	3992 (8.00)	5.4 (5.3–5.6)
NH White	43,366 (86.88)	5.7 (5.7–5.8)
Hispanic or Latino	1604 (3.21)	2.8 (2.7–2.9)
Urbanization		
Metropolitan	38,127 (76.38)	5 (5–5.1)
Nonmetropolitan	11,790 (23.62)	7.1 (7–7.3)
Place of death⁎		
Medical facility	17,963 (35.99)	–
Decedent's home	13,586 (27.22)	–
Hospice facility	1901 (3.81)	–
Nursing home/long-term care facility	14,301 (28.65)	–
Others	2056 (4.12)	–
Unknown	69 (0.14)	–

NH: non-Hispanic.

⁎ Age Adjusted Mortality Rates (AAMRs) are not applicable for place of death and are not available for NH American Indian or Alaska Natives.

### 3.1 Annual trends for HF and CRC-related age-adjusted mortality rate (AAMR)

**Fig. 1:**
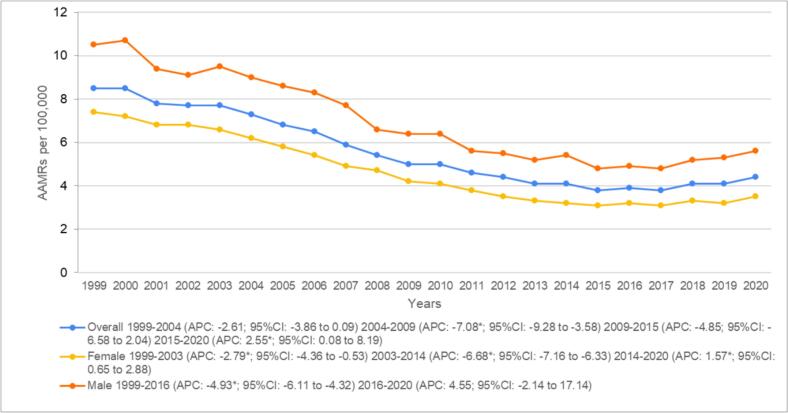
Overall and sex-stratified heart failure and colon cancer related AAMRs per 100,000 in adults aged 65 and above in the United States, 1999 to 2020. *Indicates that the annual percentage change (APC) is significantly different from zero at α = 0.05.

Overall, AAMRs for older patients with heart failure and concomitant colon cancer remained stable in the initial years, showing little change from 8.5 (95 % CI: 8.2–8.9) in 1999 to 7.3 (95 % CI: 7–7.5) in 2004 (APC: −2.61; 95 % CI: −3.86 to 0.09). This was followed by a significant decrease, with rates reaching 5.0 (95 % CI: 4.8–5.2) in 2009 (APC: −7.08*; 95 % CI: −9.28 to −3.58). Another period of stability soon followed until 2015, with AAMRs at 3.8 (95 % CI: 3.6–4) (APC: −4.85; 95 % CI: −6.58 to 2.04). In later years, a significant uptick was seen, with AAMRs reaching 4.4 (95 % CI: 4.2–4.6) by 2020 (APC: 2.55*; 95 % CI: 0.08 to 8.19). (Fig. 1 and Supplementary Tables 3 and 4).

### 3.2 HF and CRC-related AAMR stratified by sex

When analyzing mortality by gender, males were found to have notably higher AAMRs than females throughout the study period (total AAMR male: 6.7; 95 % CI: 6.6–6.8 vs total AAMR female: 4.5; 95 % CI: 4.5–4.6) with rates decreasing significantly from 10.5 (95 % CI: 9.9–11.1) to 4.9 (95 % CI: 4.6–5.2) between 1999 and 2016 respectively (APC: −4.93*; 95 % CI: −6.11 to −4.32) and consequently showing little variation to 5.6 (95 % CI: 5.3–5.9) in 2020 (APC: 4.55; 95 % CI: −2.14 to 17.14). Similarly, females also presented a significant decrease in the initial years with AAMRs falling from 7.4 (95 % CI: 7.1–7.8) to 6.6 (95 % CI: 6.3–6.9) between 1999 and 2003 (APC: −2.79*; 95 % CI: −4.36 to −0.53) with a further reduction to 3.2 (95 % CI: 3.0–3.4) by 2014 noted (APC: −6.68*; 95 % CI: −7.16 to −6.33). In contrast, in females, a significant increase in mortality was evident towards the end of the study period, with death rates inflating to 3.5 (95 % CI: 3.3–3.7) by 2020. (APC: 1.57*; 95 % CI: 0.65 to 2.88). (Fig. 1 and Supplementary Tables 3 and 4).

### 3.3 HF and CRC-related AAMR stratified by race

**Fig. 2:**
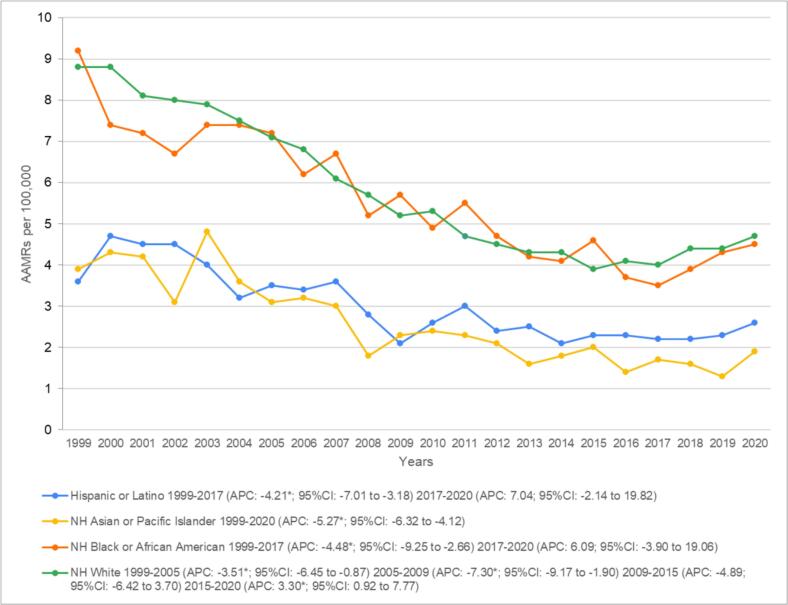
Heart failure and colon cancer-related AAMRs per 100,000 stratified by race in adults aged 65 and above in the United States, 1999 to 2020. *Indicates that the annual percentage change (APC) is significantly different from zero at α = 0.05.

Stratifying by race, AAMRs were highest in Non-Hispanic (NH) Whites (AAMR: 5.7; 95 % CI: 5.7–5.8), followed by NH Blacks/African Americans (AAMR: 5.4; 95 % CI: 5.3–5.6), Hispanics/Latinos (AAMR: 2.8; 95 % CI: 2.7–2.9), and NH Asians/Pacific Islanders (AAMR: 2.3; 95 % CI: 2.1–2.4). Similar to the overall trends, NH Whites showed a decrease in mortality between 1999 and 2005 (APC: −3.51*; 95 % CI: −6.45 to −0.87) until 2009 (APC: −7.30*; 95 % CI: −9.18 to −1.90). Rates remained stable until 2015 (APC: −4.89; 95 % CI: −6.42 to 3.70), with a surge in deaths by 2020 (3.30*; 95 % CI: 0.92 to 7.78). Mortality rates decreased until 2017 (APC: −4.49*; 95 % CI: −9.25 to −2.66) for NH Blacks/African Americans, with a non-significant increase until 2020 (APC: 6.09; 95 % CI: −3.91 to 19.07). A similar downward trend until 2017 (APC: −4.21*; 95 % CI: −7.01 to −3.18) and insignificant change until 2020 (APC: 7.04; 95 % CI: −2.14 to 19.82) were also evident in the Hispanic/Latino population. NH Asian/Pacific Islanders showed a continuous decrease in AAMRs (APC: −5.27*; 95 % CI: −6.32 to −4.12) (Fig. 2 and Supplemental Tables 3 and 5).

### 3.4 HF and CRC-related AAMR stratified by geography

**Fig. 3:**
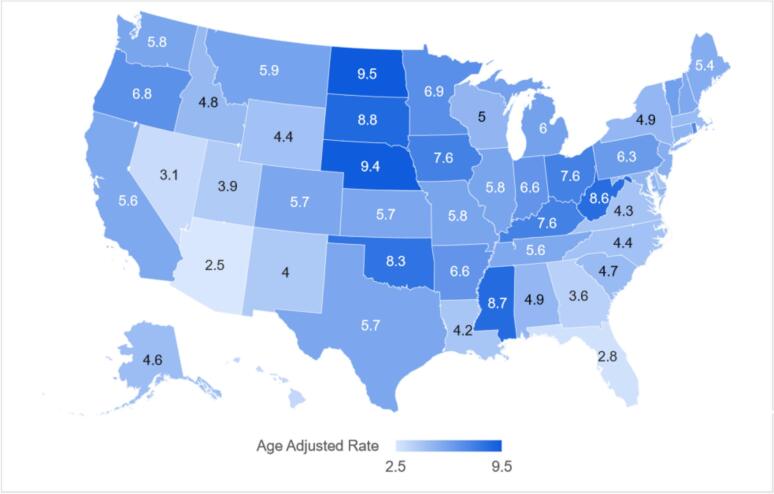
Heart failure and colon cancer-related AAMRs per 100,000 stratified by state in adults aged 65 and above in the United States, 1999 to 2020.

**Fig. 4:**
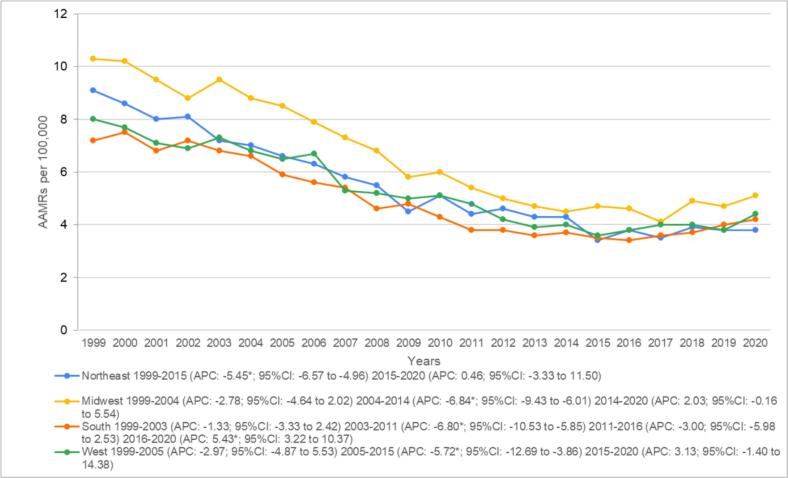
Heart failure and colon cancer-related AAMRs per 100,000 stratified by census region in adults aged 65 and above in the United States, 1999 to 2020. *Indicates that the annual percentage change (APC) is significantly different from zero at α = 0.05.

Evaluating state-wise data showed AAMRs ranging from 2.5 (95 % CI: 2.3–2.8) in Arizona to 9.5 (95 % CI: 8.3–10.7) in North Dakota with states in the top 90th percentile (West Virginia, Mississippi, South Dakota, Nebraska and North Dakota) having over triple the mortality rates than those in the lower 10th percentile (Arizona, Florida, Nevada, Hawaii and District of Columbia) (Fig. 3 and Supplemental Table 6). According to census regions, the Midwest presented with the highest overall AAMR at 6.5 (95 % CI: 6.4–6.6) followed by the Northeast at 5.4 (95 % CI: 5.3–5.5), the West at 5.2 (95 % CI: 5.1–5.3) and the South at 4.8 (95 % CI: 4.7–4.9) (Fig. 4, Supplemental Table 7).

**Fig. 5:**
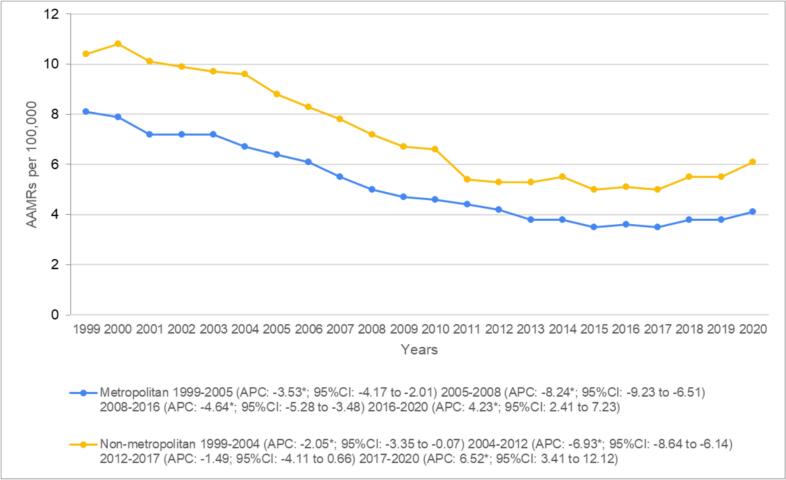
Heart failure and colon cancer-related AAMRs per 100,000 stratified by urbanization in adults aged 65 and above in the United States, 1999 to 2020. *Indicates that the annual percentage change (APC) is significantly different from zero at α = 0.05.

Annually, non-metropolitan areas had consistently higher AAMRs than metropolitan areas, with overall values of 7.1 (95 % CI: 7.0–7.3) and five (95 % CI: 5.0–5.1), respectively. Mortality rates significantly declined in the initial seventeen years in metropolitan areas from 8.1 (95 % CI: 7.8–8.4) in 1999 to 6.4 (95 % CI: 6.1–6.7) in 2005 (APC: −3.53*; 95 % CI: −4.17 to −2.01) to 5.0 (95 % CI: 4.8–5.3) in 2008 (APC: −8.24*; 95 % CI: −9.23 to −6.51) till 3.6 (95 % CI: 3.4–3.8) in 2016 (APC: −4.64*; 95 % CI: −5.28 to −3.48). This was followed by a drastic increase in death rates in the later years of the study, with AAMRs reaching 4.1 (95 % CI: 3.9–4.2) by 2020 (APC: 4.23*; 95 % CI: 2.41 to 7.24). Similarly, an initial trend of declining AAMRs was also evident in non-metropolitan areas, with mortality rates falling significantly from 10.4 (95 % CI: 9.6–11.1) in 1999 to 9.6 (95 % CI: 8.8–10.3) in 2004 (APC-2.05*; 95 % CI: −3.35 to −0.07) to 5.3 (95 % CI: 4.8–5.8) in 2012 (APC = −6.93*; 95 % CI: −8.64 to −6.14). This was followed by a non-significant decrease to 5.0 (95 % CI: 4.6–5.5) in 2017 (APC: −1.49; 95 % CI: −4.11 to 0.67). Compared with metropolitan areas, nonmetropolitan areas also saw a drastic surge in deaths towards the tail end of the study, with AAMRs reaching 6.1 (95 % CI: 5.6–6.6) in 2020. (APC: 6.52*; 95 % CI: 3.41 to 12.12) (Fig. 5 and Supplemental Tables 3 and 8).

## 4 Discussion

Our study offers a comprehensive analysis of age-adjusted mortality rates (AAMRs) for HF and CRC among individuals aged ≥65 years in the United States from 1999 to 2020. The overall AAMR across this population was 5.4 per 100,000 individuals (95 % CI: 5.4–5.5). Initially, the AAMRs remained relatively stable, beginning at 8.5 (95 % CI: 8.2–8.9) in 1999 and showed little change up to 7.3 (95 % CI: 7–7.5) in 2004. The mortality trend during this period, as indicated by an annual percentage change (APC) of −2.61 (95 % CI: − 3.86 to 0.09), was not statistically significant. A notable decline in the mortality rate was observed from 1999 to 2009, with rates decreasing to 5.0 (95 % CI: 4.8–5.2) by 2009 (APC: –7.08; 95 % CI: −9.28 to −3.59). This decline was followed by a period of stability until 2015, during which the AAMRs were approximately 3.8 (95 % CI: 3.6–4) (APC: −4.85; 95 % CI: −6.58 to 2.04), indicating a plateau in improvements. However, from 2015 to 2020, a concerning reversal was observed as AAMRs increased to 4.4 (95 % CI: 4.2–4.6) (APC: 2.55; 95 % CI: 0.09 to 8.19), highlighting an emerging public health challenge.

These findings align with previous research. For instance, Benjamin et al. reported a plateau and eventual reversal in HF mortality trends among older adults in the U.S., particularly in non-metropolitan areas and among racially diverse populations [13]. Similarly, analysis from the Surveillance, Epidemiology, and End Results (SEER) program and CDC reports noted that while CRC mortality decreased significantly due to early screening efforts, recent data suggest plateaus or slight increases among certain subpopulations, especially younger adults and some racial minorities [14].

Sex stratification indicated that males consistently exhibited higher AAMRs than females over the research period (6.7 vs. 4.5 per 100,000, respectively). While both groups saw declines in mortality, the reduction was more pronounced in males. Notably, mortality rates for females increased slightly towards the end of the study period, suggesting possible discrepancies that warrant further investigation. These findings mirror those of Virani et al., who reported sex-specific disparities in cardiovascular mortality, likely due to diagnostic bias, underreporting of symptoms, and differences in access to appropriate care [15].

Racial and geographical disparities were also observed. Non-Hispanic Whites had the highest overall AAMRs, followed by non-Hispanic Blacks, Hispanics/Latinos, and non-Hispanic Asians/Pacific Islanders. Interestingly, non-Hispanic whites experienced a sharp increase in death rates in the latter half of the research period, whereas non-Hispanic Asian/Pacific Islanders showed a steady decline. These findings are in line with Islami et al., who found that racial disparities in cancer and cardiovascular disease outcomes were often linked to differences in socioeconomic status, healthcare access, education, and preventive screening behaviors rather than genetics [[16], [17], [18]].

Geographically, AAMRs were generally higher in non-metropolitan areas than in metropolitan areas; within the census regions, the Midwest had the highest rates. These findings reflect conclusions from the CDC's Rural Health Report, which noted that rural residents face significant health disparities, including later-stage diagnoses and limited access to care [19].

The integrated analysis of HF with CRC is a methodological approach that contrasts with previous studies that have examined these conditions in isolation [20,21]. Our decision to analyze them concurrently was guided by emerging evidence suggesting potential interconnections. Chronic systemic inflammation, a characteristic feature of CRC, is implicated in the pathogenesis and progression of HF. Moreover, certain cancer therapies (e.g., anthracycline, chemotherapies and radiation) are known to have cardiotoxic effects [7,8,22], complicating the clinical management of patients with comorbid HF and CRC [22]. Multidisciplinary management models, including cardio-oncology clinics and patient-centric care frameworks that integrate oncologists and cardiologists, are emerging to address this dual burden [23].

The global prevalence of heart failure (HF) is estimated to affect 64 million individuals, with an increasing incidence in numerous countries, including the United States [24]. The “Get With The Guidelines-Heart Failure” (GWTG-HF) registry, which encompasses 39,982 patients hospitalized for HF in the US, reported a 5-year mortality rate of 75 % [25]. Projections by Virani et al. also indicate that the prevalence of HF is expected to increase by approximately 46 % from 2012 to 2030, underscoring a significant public health concern [15]. A study evaluating long-term trends, conducted by Loh et al., corroborates our findings regarding the mortality trend associated with heart failure, demonstrating a decline in 3-year all-cause mortality among patients with HFrEF, from 36.4 % in 1993–1998 to 31.5 % in 2005–2010 (*p* = 0.022) [26].

Colorectal cancer remains the second most deadly cancer globally, responsible for an estimated 881,000 deaths in 2018, representing 5.8 % of all cancer-related fatalities and by 2030, it is projected that CRC will cause 1.1 million deaths annually and 2.2 million new cases globally, increasing the overall disease burden by 60 % [27]. Additionally in the U.S., CRC incidence and mortality rates vary significantly across racial groups, with disparities often attributed to modifiable social determinants of health, such as diet, screening practices, healthcare access, financial barriers, and education [16,17]. These rising trends are associated with demographic transitions, westernized diets, environmental factors, and sedentary lifestyles, particularly in low- to medium-Human Development Index (HDI) countries [28].

Our combined analyses highlight substantial intersections between HF and CRC, revealing synergistic patterns in mortality trends that might otherwise be overlooked in isolated studies. The observed disparities across sex, race, and geography underscore the need for targeted public health interventions, such as increased screening in high-risk areas, education campaigns tailored by demographics, and improved access to preventive care in rural communities. More broadly, this integrated approach emphasizes the value of coordinated risk assessment and holistic care models. Future policies and clinical guidelines should consider the development of shared surveillance frameworks and multidisciplinary care pathways to more effectively manage overlapping chronic conditions in aging populations.

### 4.1 Limitations

Although this study provides valuable insights into the rising mortality rates associated with colon cancer and heart failure, several limitations must be acknowledged. First, the death certificates in the CDC WONDER database, which serves as a primary data source, may be subject to human error. Additionally, a potential bias in the analysis of geographic mortality trends may arise due to the misclassification of certain places of death for a small percentage of the population. The absence of detailed information on socioeconomic status, comorbid conditions, and access to healthcare is another factor that could affect the authenticity of mortality outcomes. Furthermore, due to insufficient data, an annual trend analysis of NH Native Hawaiians and American Indians was not feasible. However, to ensure the accuracy of the mortality rates, data from this group were included in a broader analysis of other categories. Finally, this study was confined to a specific age group in the United States (65 years or older), and the findings may not apply to younger populations or other countries. Comparative studies analyzing similar trends across other countries may help identify the best practices and innovative approaches to improve outcomes for older adults globally.

## 5 Conclusion

The subsequent plateau following 2009, followed by a rise in mortality rates, necessitates renewed efforts to sustain progress and address emerging challenges. Given the increasing trends in mortality in recent years, future research should explore the socioeconomic determinants of mortality to understand how education, healthcare accessibility, and income levels contribute to the disparities in AAMRs. Other areas warranting exploration include environmental changes, diet, and technological advancements in the treatment processes. Additionally, investigating other chronic conditions, such as diabetes or chronic obstructive pulmonary disease (COPD), in older adults may provide a more comprehensive understanding of the factors influencing longevity.

## CRediT authorship contribution statement

**Hafsah Alim Ur Rahman:** Formal analysis, Data curation, Conceptualization. **Nimrah Iqbal:** Supervision, Software, Resources. **Muhammad Ahmed Ali Fahim:** Writing – original draft, Visualization, Data curation. **Fayza Salman:** Methodology, Investigation, Formal analysis. **Syed Hassan Ahmed:** Visualization, Resources, Methodology. **Omama Asim:** Writing – original draft, Investigation, Data curation. **Taha Mansoor:** Writing – review & editing, Supervision, Project administration. **Muhammad Zain Farooq:** Writing – review & editing, Validation, Formal analysis. **Muhammad Sohaib Asghar:** Writing – review & editing, Supervision, Project administration, Conceptualization.

## Ethical approval

This study uses publicly available data and does not require ethical approval.

## Declaration of competing interest

The authors declare that they have no known competing financial interests or personal relationships that could have appeared to influence the work reported in this paper.

## Appendix A Supplementary data

Supplementary tables Image 1

## References

[1] Emmons-Bell S., Johnson C., Roth G. (2022 Aug 11). Prevalence, incidence and survival of heart failure: a systematic review. Heart.

[2] Jaaks P., Coker E.A., Vis D.J., Edwards O., Carpenter E.F., Leto S.M., Dwane L., Sassi F., Lightfoot H., Barthorpe S., van der Meer D., Yang W., Beck A., Mironenko T., Hall C., Hall J., Mali I., Richardson L., Tolley C., Morris J., Thomas F., Lleshi E., Aben N., Benes C.H., Bertotti A., Trusolino L., Wessels L., Garnett M.J. (2022 Mar). Effective drug combinations in breast, colon and pancreatic cancer cells. Nature.

[3] McMurray J.J.V., Solomon S.D., Inzucchi S.E., Køber L., Kosiborod M.N., Martinez F.A., Ponikowski P., Sabatine M.S., Anand I.S., Bělohlávek J., Böhm M., Chiang C.E., Chopra V.K., de Boer R.A., Desai A.S., Diez M., Drozdz J., Dukát A., Ge J., Howlett J.G., Katova T., Kitakaze M., Ljungman C.E.A., Merkely B., Nicolau J.C., O’Meara E., Petrie M.C., Vinh P.N., Schou M., Tereshchenko S., Verma S., Held C., DeMets D.L., Docherty K.F., Jhund P.S., Bengtsson O., Sjöstrand M., Langkilde A.M., DAPA-HF Trial Committees and Investigators (2019). Dapagliflozin in patients with heart failure and reduced ejection fraction. N. Engl. J. Med..

[4] Heidenreich P.A., Bozkurt B., Aguilar D., Allen L.A., Byun J.J., Colvin M.M., Deswal A., Drazner M.H., Dunlay S.M., Evers L.R., Fang J.C., Fedson S.E., Fonarow G.C., Hayek S.S., Hernandez A.F., Khazanie P., Kittleson M.M., Lee C.S., Link M.S., Milano C.A., Nnacheta L.C., Sandhu A.T., Stevenson L.W., Vardeny O., Vest A.R., Yancy C.W. (2022 May 3). 2022 AHA/ACC/HFSA guideline for the management of heart failure: a report of the American College of Cardiology/American Heart Association Joint Committee on Clinical Practice Guidelines. Circulation.

[5] Bozkurt B., Ahmad T., Alexander K.M. (2023). Heart failure epidemiology and outcomes statistics: a report of the Heart Failure Society of America. J. Card. Fail..

[6] Bray F., Ferlay J., Soerjomataram I., Siegel R.L., Torre L.A., Jemal A. (2020 Jul). Global cancer statistics 2018: GLOBOCAN estimates of incidence and mortality worldwide for 36 cancers in 185 countries. CA Cancer J. Clin. 2018 Nov;68(6):394–424. doi: 10.3322/caac.21492. Epub 2018 Sep 12. Erratum in. CA Cancer J. Clin..

[7] Zhang C., Cheng Y., Luo D., Wang J., Liu J., Luo Y., Zhou W., Zhuo Z., Guo K., Zeng R., Yang J. (2021 Mar 18). Association between cardiovascular risk factors and colorectal cancer: a systematic review and meta-analysis of prospective cohort studies. eClinicalMedicine.

[8] Li C., Ngorsuraches S., Chou C., Chen L., Qian J. (2021). Risk factors of fluoropyrimidine induced cardiotoxicity among cancer patients: a systematic review and meta-analysis. Crit. Rev. Oncol. Hematol..

[9] Snyder J.E., Upton R.D., Hassett T.C., Lee H., Nouri Z., Dill M. (2023 Apr 3). Black representation in the primary care physician workforce and its association with population life expectancy and mortality rates in the US. JAMA Netw. Open.

[10] Centers for Disease Control and Prevention (2024). About Multiple Cause of Death, 1999–2020. https://wonder.cdc.gov/mcd-icd10.html.

[11] Ingram Deborah D., Franco Sheila J. (2014). 2013 NCHS urban-rural classification scheme for counties. Vital Health Stat. Ser. 2 Data Eval. Methods Res..

[12] Centers for Disease Control and Prevention (July 24, 2024). https://www.cdc.gov/nchs/hus/sources-definitions/geographic-region.htm.

[13] Benjamin E.J., Muntner P., Alonso A. (2020). Heart disease and stroke statistics—2020 update: a report from the American Heart Association. Circulation.

[14] Siegel R.L., Miller K.D., Goding Sauer A. (2020). Colorectal cancer statistics, 2020. CA Cancer J. Clin..

[15] Virani S.S., Alonso A., Aparicio H.J. (2021). Heart disease and stroke statistics—2021 update. Circulation.

[16] Irby K., Anderson W.F., Henson D.E., Devesa S.S. (2006). Emerging and widening colorectal carcinoma disparities between Blacks and Whites in the United States (1975–2002). Cancer Epidemiol. Biomarkers Prev..

[17] Lansdorp-Vogelaar I., Kuntz K.M., Knudsen A.B. (2012). Contribution of screening and survival differences to racial disparities in colorectal cancer rates. Cancer Epidemiol. Biomarkers Prev..

[18] Islami F., Ward E.M., Sung H. (2021). Annual report to the nation on the status of cancer, part 1: national cancer statistics. J. Natl. Cancer Inst..

[19] Centers for Disease Control and Prevention (CDC) (2023). Rural Health Report.

[20] Kusnik A., Renjithlal S.L.M., Chodos A., Shanmukhappa S.C., Eid M.M., Renjith K.M. (2023 Jul 12). Trends in colorectal cancer mortality in the United States, 1999–2020. Gastroenterol. Res..

[21] Siddiqi T.J., AMK Minhas, Greene S.J., HGC Van Spall, Khan S.S., Pandey A. (2022 Nov). Trends in heart failure-related mortality among older adults in the United States from 1999 to 2019. JACC Heart Fail..

[22] Armenian S.H., Xu L., Ky B. (2016). Cardiovascular disease among survivors of adult-onset cancer: a community-based retrospective cohort study. J. Clin. Oncol..

[23] Curigliano G., Lenihan D., Fradley M. (2020). Management of cardiac disease in cancer patients throughout oncological treatment: ESMO consensus recommendations. Ann. Oncol..

[24] GBD 2017 Disease and Injury Incidence and Prevalence Collaborators (2018). Global, regional, and national incidence, prevalence, and years lived with disability for 354 diseases and injuries for 195 countries and territories, 1990–2017: a systematic analysis for the Global Burden of Disease Study 2017. Lancet.

[25] Steinberg B.A., Zhao X., Heidenreich P.A. (2012). Trends in patients hospitalized with heart failure and preserved left ventricular ejection fraction: prevalence, therapies, and outcomes. Circulation.

[26] Loh J.C., Creaser J., Rourke D.A., Livingston N., Harrison T.K., Vandenbogaart E., Moriguchi J., Hamilton M.A., Tseng C.H., Fonarow G.C., Horwich T.B. (2013 May). Temporal trends in treatment and outcomes for advanced heart failure with reduced ejection fraction from 1993–2010: findings from a university referral center. Circ. Heart Fail..

[27] Rawla P., Sunkara T., Barsouk A. (2019). Epidemiology of colorectal cancer: incidence, mortality, survival, and risk factors. Prz. Gastroenterol..

[28] Arnold M., Sierra M.S., Laversanne M. (2017). Global patterns and trends in colorectal cancer incidence and mortality. Gut.

