# Supplementary material for: Mortality trends in heart failure and colon cancer: Insights into gender, ethnic, and regional disparities in the United States (1999–2020)
Source: Am Heart J Plus. 2025 Dec 16;61:100699. doi: 10.1016/j.ahjo.2025.100699 (PMC12925751; doi:10.1016/j.ahjo.2025.100699)
Supplement: Supplementary file 1 — Supplementary tables [file mmc1.docx]

**SUPPLEMENTARY FILES:**

**Supplemental Table 1:** Heart Failure and Colon Cancer related Deaths, Stratified by Sex and Race, in Older Adults in the United States, 1999 to 2020

| **Deaths** | | | | | | | | | |
| --- | --- | --- | --- | --- | --- | --- | --- | --- | --- |
| **Year** | **Overall** | **Women** | **Men** | **NH White** | **NH Black or African American** | **NH Asian or Pacific Islander** | **NH American Indian or Alaska Native** | **Hispanic or Latino** | **Population** |
| 1999 | 2930 | 1662 | 1268 | 2604 | 240 | 24 | - | 47 | 34797841 |
| 2000 | 2942 | 1643 | 1299 | 2636 | 198 | 27 | - | 67 | 34991753 |
| 2001 | 2742 | 1571 | 1171 | 2441 | 193 | 32 | - | 67 | 35290291 |
| 2002 | 2750 | 1575 | 1175 | 2458 | 181 | 26 | - | 70 | 35522207 |
| 2003 | 2781 | 1539 | 1242 | 2455 | 203 | 40 | - | 68 | 35863529 |
| 2004 | 2666 | 1469 | 1197 | 2365 | 206 | 31 | - | 58 | 36203319 |
| 2005 | 2555 | 1384 | 1171 | 2246 | 205 | 29 | - | 66 | 36649798 |
| 2006 | 2503 | 1327 | 1176 | 2217 | 177 | 31 | - | 68 | 37164107 |
| 2007 | 2311 | 1203 | 1108 | 2001 | 198 | 33 | - | 75 | 37825711 |
| 2008 | 2171 | 1187 | 984 | 1917 | 159 | 21 | - | 61 | 38777621 |
| 2009 | 2038 | 1057 | 981 | 1770 | 179 | 29 | - | 49 | 39623175 |
| 2010 | 2066 | 1059 | 1007 | 1804 | 157 | 30 | - | 65 | 40267984 |
| 2011 | 1943 | 1023 | 920 | 1639 | 183 | 32 | - | 79 | 41394141 |
| 2012 | 1899 | 970 | 929 | 1624 | 163 | 31 | - | 69 | 43145356 |
| 2013 | 1813 | 920 | 893 | 1552 | 151 | 26 | - | 76 | 44704074 |
| 2014 | 1877 | 913 | 964 | 1613 | 154 | 31 | - | 66 | 46243211 |
| 2015 | 1780 | 879 | 901 | 1471 | 180 | 37 | - | 79 | 47760852 |
| 2016 | 1854 | 917 | 937 | 1576 | 151 | 28 | 10 | 83 | 49244195 |
| 2017 | 1854 | 909 | 945 | 1568 | 146 | 38 | 11 | 88 | 50858679 |
| 2018 | 2049 | 1004 | 1045 | 1740 | 169 | 37 | - | 89 | 52431193 |
| 2019 | 2085 | 987 | 1098 | 1752 | 187 | 31 | 12 | 97 | 54058263 |
| 2020 | 2308 | 1104 | 1204 | 1917 | 212 | 50 | 11 | 117 | 55659365 |
| **Total** | 49917 | 26302 | 23615 | 43366 | 3992 | 694 | 167 | 1604 | 928476665 |

**Supplemental Table 2:** Heart Failure and Colon Cancer related Mortality, Stratified by Place of Death in Older Adults in the United States, 1999 to 2020

| **Deaths** | | | | | | |
| --- | --- | --- | --- | --- | --- | --- |
| **Year** | **Medical Facility** | **Nursing Home** | **Hospices** | **Home** | **Other** | **Unknown** |
|  |  |  |  |  |  |  |
| 1999 | 1304 | 949 | - | 578 | 98 | - |
| 2000 | 1301 | 913 | - | 636 | 90 | - |
| 2001 | 1191 | 859 | - | 607 | 83 | - |
| 2002 | 1164 | 861 | - | 609 | 114 | - |
| 2003 | 1182 | 852 | - | 626 | 111 | - |
| 2004 | 1098 | 818 | 12 | 597 | 136 | - |
| 2005 | 986 | 817 | 26 | 624 | 99 | - |
| 2006 | 1009 | 731 | 34 | 630 | 89 | 10 |
| 2007 | 861 | 658 | 70 | 603 | 111 | - |
| 2008 | 778 | 660 | 62 | 556 | 94 | 21 |
| 2009 | 701 | 545 | 83 | 572 | 99 | 38 |
| 2010 | 703 | 588 | 81 | 589 | 103 | - |
| 2011 | 648 | 534 | 120 | 539 | 101 | - |
| 2012 | 596 | 517 | 122 | 593 | 70 | - |
| 2013 | 587 | 491 | 118 | 526 | 90 | - |
| 2014 | 534 | 533 | 146 | 575 | 89 | - |
| 2015 | 514 | 476 | 170 | 560 | 59 | - |
| 2016 | 535 | 474 | 153 | 618 | 74 | - |
| 2017 | 534 | 501 | 163 | 580 | 75 | - |
| 2018 | 572 | 537 | 167 | 690 | 83 | - |
| 2019 | 571 | 505 | 171 | 751 | 87 | - |
| 2020 | 594 | 482 | 203 | 927 | 101 | - |
| **Total** | 17963 | 14301 | 1901 | 13586 | 2056 | 69 |

**Supplemental Table 3:** Annual percent change (APC) of Heart Failure and Colon Cancer related Age-Adjusted Mortality Rates per 100,000 in Older Adults in the United States, 1999 to 2020

| **Year Interval** | **APC (95% CI)** |
| --- | --- |
| **Overall** | |
| 1999-2004 | -2.6115 (-3.8601 to 0.0909) |
| 2004-2009 | -7.0802* (-9.2813 to -3.5845) |
| 2009-2015 | -4.8472 (-6.5828 to 2.0358) |
| 2015-2020 | 2.5458* (0.0772 to 8.1923) |
| **Male** | |
| 1999-2016 | -4.9337* (-6.1148 to -4.3198) |
| 2016-2020 | 4.5484 (-2.136 to 17.1439) |
| **Female** | |
| 1999-2003 | -2.7926* (-4.3625 to -0.5256) |
| 2003-2014 | -6.6768* (-7.157 to -6.3266) |
| 2014-2020 | 1.5740* (0.6534 to 2.8814) |
| **NH White** | |
| 1999-2005 | -3.5075* (-6.4457 to -0.8677) |
| 2005-2009 | -7.3000* (-9.1692 to -1.8953) |
| 2009-2015 | -4.8921 (-6.4208 to 3.7027) |
| 2015-2020 | 3.3033* (0.9242 to 7.7697) |
| **NH Black or African American** | |
| 1999-2017 | -4.4760* (-9.2486 to -2.6637) |
| 2017-2020 | 6.0928 (-3.8957 to 19.0572) |
| **NH Asian or Pacific Islander** | |
| 1999-2020 | -5.2687* (-6.323 to -4.123) |
| **Hispanic or Latino** | |
| 1999-2017 | -4.2068* (-7.0109 to -3.1773) |
| 2017-2020 | 7.039 (-2.1379 to 19.8191) |
| **Nonmetropolitan areas** | |
| 1999-2004 | -2.0505* (-3.3527 to -0.0748) |
| 2004-2012 | -6.9292* (-8.6384 to -6.1414) |
| 2012-2017 | -1.4932 (-4.1118 to 0.6561) |
| 2017-2020 | 6.5173* (3.4084 to 12.1167) |
| **Metropolitan area** | |
| 1999-2005 | -3.5274* (-4.17 to -2.0073) |
| 2005-2008 | -8.2439* (-9.2255 to -6.5054) |
| 2008-2016 | -4.6386* (-5.2803 to -3.4764) |
| 2016-2020 | 4.2349* (2.4076 to 7.2349) |
| **Northeast region** | |
| 1999-2015 | -5.4532* (-6.5653 to -4.9565) |
| 2015-2020 | 0.4609 (-3.3277 to 11.5001) |
| **Midwest region** | |
| 1999-2004 | -2.7776 (-4.6376 to 2.018) |
| 2004-2014 | -6.8417* (-9.4282 to -6.0083) |
| 2014-2020 | 2.0304 (-0.1639 to 5.5362) |
| **South region** | |
| 1999-2003 | -1.33 (-3.332 to 2.4166) |
| 2003-2011 | -6.7964* (-10.5254 to -5.847) |
| 2011-2016 | -2.9976 (-5.9782 to 2.5332) |
| 2016-2020 | 5.4333* (3.2162 to 10.3738) |
| **West region** | |
| 1999-2005 | -2.966 (-4.8727 to 5.5346) |
| 2005-2015 | -5.7221* (-12.6932 to -3.861) |
| 2015-2020 | 3.1271 (-1.3969 to 14.3784) |

APC = annual percent change; NH = non-Hispanic; * Indicates that the annual percentage change (APC) is significantly different from zero at α = 0.05. AAMR = age-adjusted mortality rate

**Supplemental Table 4:** Overall and Sex‐Stratified Heart Failure and Colon Cancer-related Age-Adjusted Mortality Rates per 100,000 in Older Adults in the United States, 1999 to 2020

| **Age-Adjusted Rate (95% CI)** | | | |
| --- | --- | --- | --- |
| **Year** | **Male** | **Female** | **Overall** |
| 1999 | 10.5 (9.9 - 11.1) | 7.4 (7.1 - 7.8) | 8.5 (8.2 - 8.9) |
| 2000 | 10.7 (10.1 - 11.3) | 7.2 (6.8 - 7.5) | 8.5 (8.1 - 8.8) |
| 2001 | 9.4 (8.9 - 9.9) | 6.8 (6.5 - 7.2) | 7.8 (7.5 - 8) |
| 2002 | 9.1 (8.6 - 9.7) | 6.8 (6.5 - 7.2) | 7.7 (7.4 - 8) |
| 2003 | 9.5 (8.9 - 10) | 6.6 (6.3 - 6.9) | 7.7 (7.4 - 8) |
| 2004 | 9 (8.5 - 9.5) | 6.2 (5.9 - 6.5) | 7.3 (7 - 7.5) |
| 2005 | 8.6 (8.1 - 9.1) | 5.8 (5.5 - 6.1) | 6.8 (6.6 - 7.1) |
| 2006 | 8.3 (7.9 - 8.8) | 5.4 (5.1 - 5.7) | 6.5 (6.3 - 6.8) |
| 2007 | 7.7 (7.2 - 8.1) | 4.9 (4.6 - 5.2) | 5.9 (5.7 - 6.2) |
| 2008 | 6.6 (6.2 - 7) | 4.7 (4.4 - 4.9) | 5.4 (5.2 - 5.7) |
| 2009 | 6.4 (6 - 6.8) | 4.2 (3.9 - 4.4) | 5 (4.8 - 5.2) |
| 2010 | 6.4 (6 - 6.8) | 4.1 (3.8 - 4.3) | 5 (4.8 - 5.2) |
| 2011 | 5.6 (5.3 - 6) | 3.8 (3.6 - 4.1) | 4.6 (4.3 - 4.8) |
| 2012 | 5.5 (5.2 - 5.9) | 3.5 (3.3 - 3.8) | 4.4 (4.2 - 4.6) |
| 2013 | 5.2 (4.8 - 5.5) | 3.3 (3.1 - 3.6) | 4.1 (3.9 - 4.3) |
| 2014 | 5.4 (5.1 - 5.8) | 3.2 (3 - 3.4) | 4.1 (3.9 - 4.3) |
| 2015 | 4.8 (4.5 - 5.2) | 3.1 (2.9 - 3.3) | 3.8 (3.6 - 4) |
| 2016 | 4.9 (4.6 - 5.2) | 3.2 (3 - 3.4) | 3.9 (3.7 - 4.1) |
| 2017 | 4.8 (4.5 - 5.1) | 3.1 (2.9 - 3.3) | 3.8 (3.6 - 4) |
| 2018 | 5.2 (4.8 - 5.5) | 3.3 (3.1 - 3.5) | 4.1 (3.9 - 4.3) |
| 2019 | 5.3 (5 - 5.6) | 3.2 (3 - 3.4) | 4.1 (3.9 - 4.2) |
| 2020 | 5.6 (5.3 - 5.9) | 3.5 (3.3 - 3.7) | 4.4 (4.2 - 4.6) |
| **Total** | **6.7 (6.6 - 6.8)** | **4.5 (4.5 - 4.6)** | **5.4 (5.4 - 5.5)** |

**Supplemental Table 5:** Race‐Stratified Heart Failure and Colon Cancer-related Age-Adjusted Mortality Rates per 100,000 in Older Adults in the United States, 1999 to 2020

| **Age-Adjusted Rate (95% CI)** | | | | |
| --- | --- | --- | --- | --- |
| **Year** | **NH White** | **NH Black or African American** | **Hispanic or Latino** | **NH Asian or Pacific Islander** |
| 1999 | 8.8 (8.4 - 9.1) | 9.2 (8 - 10.3) | 3.6 (2.6 - 4.8) | 3.9 (2.4 - 5.8) |
| 2000 | 8.8 (8.5 - 9.1) | 7.4 (6.4 - 8.5) | 4.7 (3.6 - 6) | 4.3 (2.8 - 6.3) |
| 2001 | 8.1 (7.8 - 8.4) | 7.2 (6.2 - 8.2) | 4.5 (3.5 - 5.7) | 4.2 (2.8 - 5.9) |
| 2002 | 8 (7.7 - 8.4) | 6.7 (5.8 - 7.7) | 4.5 (3.5 - 5.7) | 3.1 (2 - 4.6) |
| 2003 | 7.9 (7.6 - 8.2) | 7.4 (6.4 - 8.4) | 4 (3.1 - 5.1) | 4.8 (3.4 - 6.6) |
| 2004 | 7.5 (7.2 - 7.8) | 7.4 (6.4 - 8.4) | 3.2 (2.4 - 4.2) | 3.6 (2.4 - 5.1) |
| 2005 | 7.1 (6.8 - 7.4) | 7.2 (6.2 - 8.2) | 3.5 (2.7 - 4.5) | 3.1 (2.1 - 4.5) |
| 2006 | 6.8 (6.6 - 7.1) | 6.2 (5.3 - 7.1) | 3.4 (2.6 - 4.4) | 3.2 (2.1 - 4.5) |
| 2007 | 6.1 (5.8 - 6.4) | 6.7 (5.7 - 7.6) | 3.6 (2.8 - 4.5) | 3 (2.1 - 4.2) |
| 2008 | 5.7 (5.5 - 6) | 5.2 (4.4 - 6) | 2.8 (2.1 - 3.5) | 1.8 (1.1 - 2.8) |
| 2009 | 5.2 (5 - 5.5) | 5.7 (4.9 - 6.6) | 2.1 (1.5 - 2.8) | 2.3 (1.5 - 3.3) |
| 2010 | 5.3 (5 - 5.5) | 4.9 (4.1 - 5.7) | 2.6 (2 - 3.4) | 2.4 (1.6 - 3.4) |
| 2011 | 4.7 (4.5 - 4.9) | 5.5 (4.7 - 6.3) | 3 (2.4 - 3.7) | 2.3 (1.5 - 3.2) |
| 2012 | 4.5 (4.3 - 4.7) | 4.7 (4 - 5.4) | 2.4 (1.9 - 3.1) | 2.1 (1.4 - 3) |
| 2013 | 4.3 (4 - 4.5) | 4.2 (3.5 - 4.9) | 2.5 (2 - 3.2) | 1.6 (1.1 - 2.4) |
| 2014 | 4.3 (4.1 - 4.5) | 4.1 (3.4 - 4.7) | 2.1 (1.6 - 2.6) | 1.8 (1.2 - 2.6) |
| 2015 | 3.9 (3.7 - 4.1) | 4.6 (3.9 - 5.3) | 2.3 (1.8 - 2.9) | 2 (1.4 - 2.7) |
| 2016 | 4.1 (3.9 - 4.3) | 3.7 (3.1 - 4.3) | 2.3 (1.9 - 2.9) | 1.4 (1 - 2.1) |
| 2017 | 4 (3.8 - 4.2) | 3.5 (2.9 - 4) | 2.2 (1.8 - 2.8) | 1.7 (1.2 - 2.4) |
| 2018 | 4.4 (4.2 - 4.6) | 3.9 (3.3 - 4.5) | 2.2 (1.8 - 2.7) | 1.6 (1.2 - 2.3) |
| 2019 | 4.4 (4.2 - 4.6) | 4.3 (3.6 - 4.9) | 2.3 (1.9 - 2.9) | 1.3 (0.9 - 1.9) |
| 2020 | 4.7 (4.5 - 4.9) | 4.5 (3.9 - 5.1) | 2.6 (2.1 - 3.1) | 1.9 (1.4 - 2.5) |
| **Total** | 5.7 (5.7 - 5.8) | 5.4 (5.3 - 5.6) | 2.8 (2.7 - 2.9) | 2.3 (2.1 - 2.4) |

NH: Non-Hispanic

**Supplemental Table 6:** Heart Failure and Colon Cancer related Age-Adjusted Mortality Rates per 100,000, Stratified by States in Older Adults in the United States, 1999 to 2020

| **State** | **Age-Adjusted Rate (95% CI)** |
| --- | --- |
| Alabama | 4.9 (4.5 - 5.2) |
| Alaska | 4.6 (3.3 - 6.1) |
| Arizona | 2.5 (2.3 - 2.8) |
| Arkansas | 6.6 (6 - 7.1) |
| California | 5.6 (5.5 - 5.8) |
| Colorado | 5.7 (5.2 - 6.1) |
| Connecticut | 5.1 (4.7 - 5.5) |
| Delaware | 4.3 (3.6 - 5.1) |
| District of Columbia | 3.5 (2.7 - 4.6) |
| Florida | 2.8 (2.7 - 2.9) |
| Georgia | 3.6 (3.4 - 3.9) |
| Hawaii | 3.3 (2.8 - 3.9) |
| Idaho | 4.8 (4.1 - 5.5) |
| Illinois | 5.8 (5.5 - 6) |
| Indiana | 6.6 (6.3 - 7) |
| Iowa | 7.6 (7 - 8.1) |
| Kansas | 5.7 (5.2 - 6.2) |
| Kentucky | 7.6 (7.1 - 8.1) |
| Louisiana | 4.2 (3.9 - 4.6) |
| Maine | 5.4 (4.7 - 6) |
| Maryland | 5 (4.7 - 5.4) |
| Massachusetts | 4.7 (4.4 - 5) |
| Michigan | 6 (5.7 - 6.2) |
| Minnesota | 6.9 (6.5 - 7.3) |
| Mississippi | 8.7 (8.1 - 9.4) |
| Missouri | 5.8 (5.4 - 6.1) |
| Montana | 5.9 (5.1 - 6.8) |
| Nebraska | 9.4 (8.6 - 10.2) |
| Nevada | 3.1 (2.6 - 3.5) |
| New Hampshire | 5.6 (4.9 - 6.3) |
| New Jersey | 5.3 (5 - 5.5) |
| New Mexico | 4 (3.5 - 4.5) |
| New York | 4.9 (4.7 - 5) |
| North Carolina | 4.4 (4.2 - 4.7) |
| North Dakota | 9.5 (8.3 - 10.7) |
| Ohio | 7.6 (7.3 - 7.9) |
| Oklahoma | 8.3 (7.8 - 8.9) |
| Oregon | 6.8 (6.3 - 7.2) |
| Pennsylvania | 6.3 (6.1 - 6.5) |
| Rhode Island | 7.2 (6.4 - 8.1) |
| South Carolina | 4.7 (4.3 - 5.1) |
| South Dakota | 8.8 (7.7 - 9.9) |
| Tennessee | 5.6 (5.2 - 5.9) |
| Texas | 5.7 (5.5 - 5.9) |
| Utah | 3.9 (3.4 - 4.4) |
| Vermont | 6.1 (5.1 - 7.2) |
| Virginia | 4.3 (4 - 4.6) |
| Washington | 5.8 (5.5 - 6.2) |
| West Virginia | 8.6 (7.8 - 9.3) |
| Wisconsin | 5 (4.7 - 5.3) |
| Wyoming | 4.4 (3.4 - 5.6) |

**Supplemental Table 7:** Heart Failure and Colon Cancer related -related Age-Adjusted Mortality Rates per 100,000, Stratified by Census Region in Older Adults in the United States, 1999 to 2020

| **Census Region** | **Year** | **Age-Adjusted Rate (95% CI)** |
| --- | --- | --- |
| Northeast | 1999 | 9.1 (8.4 - 9.8) |
| Northeast | 2000 | 8.6 (7.9 - 9.3) |
| Northeast | 2001 | 8 (7.4 - 8.7) |
| Northeast | 2002 | 8.1 (7.5 - 8.7) |
| Northeast | 2003 | 7.2 (6.6 - 7.8) |
| Northeast | 2004 | 7 (6.4 - 7.6) |
| Northeast | 2005 | 6.6 (6 - 7.2) |
| Northeast | 2006 | 6.3 (5.8 - 6.9) |
| Northeast | 2007 | 5.8 (5.2 - 6.3) |
| Northeast | 2008 | 5.5 (5 - 6) |
| Northeast | 2009 | 4.5 (4 - 5) |
| Northeast | 2010 | 5.1 (4.6 - 5.6) |
| Northeast | 2011 | 4.4 (4 - 4.9) |
| Northeast | 2012 | 4.6 (4.2 - 5.1) |
| Northeast | 2013 | 4.3 (3.9 - 4.7) |
| Northeast | 2014 | 4.3 (3.9 - 4.7) |
| Northeast | 2015 | 3.4 (3 - 3.7) |
| Northeast | 2016 | 3.8 (3.4 - 4.2) |
| Northeast | 2017 | 3.5 (3.1 - 3.9) |
| Northeast | 2018 | 3.9 (3.5 - 4.3) |
| Northeast | 2019 | 3.8 (3.4 - 4.2) |
| Northeast | 2020 | 3.8 (3.5 - 4.2) |
| Northeast | **Total** | 5.4 (5.3 - 5.5) |
| Midwest | 1999 | 10.3 (9.7 - 11) |
| Midwest | 2000 | 10.2 (9.6 - 10.9) |
| Midwest | 2001 | 9.5 (8.8 - 10.1) |
| Midwest | 2002 | 8.8 (8.1 - 9.4) |
| Midwest | 2003 | 9.5 (8.9 - 10.2) |
| Midwest | 2004 | 8.8 (8.2 - 9.5) |
| Midwest | 2005 | 8.5 (7.9 - 9.1) |
| Midwest | 2006 | 7.9 (7.4 - 8.5) |
| Midwest | 2007 | 7.3 (6.8 - 7.9) |
| Midwest | 2008 | 6.8 (6.3 - 7.4) |
| Midwest | 2009 | 5.8 (5.3 - 6.3) |
| Midwest | 2010 | 6 (5.5 - 6.5) |
| Midwest | 2011 | 5.4 (4.9 - 5.8) |
| Midwest | 2012 | 5 (4.6 - 5.4) |
| Midwest | 2013 | 4.7 (4.3 - 5.1) |
| Midwest | 2014 | 4.5 (4.1 - 4.9) |
| Midwest | 2015 | 4.7 (4.3 - 5.2) |
| Midwest | 2016 | 4.6 (4.2 - 5) |
| Midwest | 2017 | 4.1 (3.8 - 4.5) |
| Midwest | 2018 | 4.9 (4.5 - 5.3) |
| Midwest | 2019 | 4.7 (4.3 - 5.1) |
| Midwest | 2020 | 5.1 (4.7 - 5.5) |
| Midwest | **Total** | 6.5 (6.4 - 6.6) |
| South | 1999 | 7.2 (6.8 - 7.7) |
| South | 2000 | 7.5 (7 - 8) |
| South | 2001 | 6.8 (6.3 - 7.2) |
| South | 2002 | 7.2 (6.7 - 7.6) |
| South | 2003 | 6.8 (6.4 - 7.3) |
| South | 2004 | 6.6 (6.2 - 7.1) |
| South | 2005 | 5.9 (5.5 - 6.3) |
| South | 2006 | 5.6 (5.2 - 6.1) |
| South | 2007 | 5.4 (5 - 5.8) |
| South | 2008 | 4.6 (4.2 - 5) |
| South | 2009 | 4.8 (4.4 - 5.1) |
| South | 2010 | 4.3 (3.9 - 4.6) |
| South | 2011 | 3.8 (3.5 - 4.2) |
| South | 2012 | 3.8 (3.5 - 4.1) |
| South | 2013 | 3.6 (3.3 - 3.9) |
| South | 2014 | 3.7 (3.4 - 4) |
| South | 2015 | 3.5 (3.2 - 3.8) |
| South | 2016 | 3.4 (3.2 - 3.7) |
| South | 2017 | 3.6 (3.3 - 3.8) |
| South | 2018 | 3.7 (3.5 - 4) |
| South | 2019 | 4 (3.7 - 4.3) |
| South | 2020 | 4.2 (3.9 - 4.5) |
| South | **Total** | 4.8 (4.7 - 4.9) |
| West | 1999 | 8 (7.3 - 8.6) |
| West | 2000 | 7.7 (7.1 - 8.4) |
| West | 2001 | 7.1 (6.5 - 7.7) |
| West | 2002 | 6.9 (6.2 - 7.5) |
| West | 2003 | 7.3 (6.7 - 7.9) |
| West | 2004 | 6.8 (6.2 - 7.3) |
| West | 2005 | 6.5 (6 - 7.1) |
| West | 2006 | 6.7 (6.1 - 7.3) |
| West | 2007 | 5.3 (4.8 - 5.8) |
| West | 2008 | 5.2 (4.7 - 5.7) |
| West | 2009 | 5 (4.5 - 5.5) |
| West | 2010 | 5.1 (4.6 - 5.5) |
| West | 2011 | 4.8 (4.4 - 5.3) |
| West | 2012 | 4.2 (3.8 - 4.6) |
| West | 2013 | 3.9 (3.5 - 4.3) |
| West | 2014 | 4 (3.6 - 4.4) |
| West | 2015 | 3.6 (3.2 - 4) |
| West | 2016 | 3.8 (3.4 - 4.2) |
| West | 2017 | 4 (3.6 - 4.4) |
| West | 2018 | 4 (3.6 - 4.4) |
| West | 2019 | 3.8 (3.5 - 4.2) |
| West | 2020 | 4.4 (4.1 - 4.8) |
| West | **Total** | 5.2 (5.1 - 5.3) |
| **Total** | **Total** | 5.4 (5.4 - 5.5) |

**Supplemental Table 8:** Heart Failure and Colon Cancer related Age-Adjusted Mortality Rates per 100,000, Stratified by Urban-Rural Classification in Older Adults in the United States, 1999 to 2020

|  | | |
| --- | --- | --- |
| **Age-Adjusted Rate (95% CI)** | | |
| **Year** | **Metropolitan** | **Nonmetropolitan** |
| 1999 | 8.1 (7.8 - 8.4) | 10.4 (9.6 - 11.1) |
| 2000 | 7.9 (7.6 - 8.2) | 10.8 (10.1 - 11.6) |
| 2001 | 7.2 (6.9 - 7.5) | 10.1 (9.3 - 10.8) |
| 2002 | 7.2 (6.9 - 7.5) | 9.9 (9.2 - 10.7) |
| 2003 | 7.2 (6.9 - 7.5) | 9.7 (9 - 10.5) |
| 2004 | 6.7 (6.4 - 7) | 9.6 (8.8 - 10.3) |
| 2005 | 6.4 (6.1 - 6.7) | 8.8 (8.1 - 9.5) |
| 2006 | 6.1 (5.9 - 6.4) | 8.3 (7.6 - 9) |
| 2007 | 5.5 (5.2 - 5.8) | 7.8 (7.2 - 8.5) |
| 2008 | 5 (4.8 - 5.3) | 7.2 (6.6 - 7.8) |
| 2009 | 4.7 (4.4 - 4.9) | 6.7 (6.1 - 7.3) |
| 2010 | 4.6 (4.4 - 4.9) | 6.6 (6 - 7.2) |
| 2011 | 4.4 (4.1 - 4.6) | 5.4 (4.9 - 5.9) |
| 2012 | 4.2 (4 - 4.4) | 5.3 (4.8 - 5.8) |
| 2013 | 3.8 (3.6 - 4) | 5.3 (4.8 - 5.8) |
| 2014 | 3.8 (3.6 - 4) | 5.5 (5 - 6) |
| 2015 | 3.5 (3.3 - 3.7) | 5 (4.5 - 5.5) |
| 2016 | 3.6 (3.4 - 3.8) | 5.1 (4.6 - 5.6) |
| 2017 | 3.5 (3.4 - 3.7) | 5 (4.6 - 5.5) |
| 2018 | 3.8 (3.6 - 4) | 5.5 (5 - 6) |
| 2019 | 3.8 (3.6 - 4) | 5.5 (5 - 6) |
| 2020 | 4.1 (3.9 - 4.2) | 6.1 (5.6 - 6.6) |
| Total | 5 (5 - 5.1) | 7.1 (7 - 7.3) |
